# Effects of *Vachellia mearnsii* Tannin Extract as an Additive on Fermentation Quality, Aerobic Stability, and Microbial Modulation of Maize Silage

**DOI:** 10.3390/microorganisms11112767

**Published:** 2023-11-14

**Authors:** Thamsanqa Doctor Empire Mpanza, Sinalo Mani

**Affiliations:** 1 Animal Nutrition, Agricultural Research Council—Animal Production, Private Bag X2, Irene 0062, South Africa; 2 Department of Agriculture and Animal Health, Science Campus, University of South Africa, Private Bag X6, Florida 1710, South Africa; 3 Gastrointestinal Microbiology and Biotechnology, Agricultural Research Council—Animal Production, Private Bag X2, Irene 0062, South Africa; manis@arc.agric.za

**Keywords:** silage additives, condensed tannin, fermentative quality, bacterial modulation, silage quality

## Abstract

Maize silage is produced to alleviate the effects of forage shortages on ruminant animals, particularly during the dry season. Microorganisms play a significant role in silage fermentation and thus, to a large extent, determine the silage quality. The modulation of silage microorganisms may help to inhibit undesirable bacteria and improve the silage quality. Therefore, condensed tannin extract from *Vachellia mearnsii* bark was used as an additive in maize silage during ensiling. Hence, this study evaluated the effects of a tannin extract (condensed tannin) additive on the fermentative quality, aerobic stability, and bacterial composition of maize silage. A mini-silo experiment on maize with five treatments was conducted for 75 days. The silage treatments were as follows: (T1) maize forage with no inoculation (negative control); (T2) maize forage inoculated with LAB and 1% tannin extract; (T3) maize forage inoculated with LAB only (positive control); (T4) and maize forage inoculated with LAB and 2% tannin extract; (T5) maize forage inoculated with LAB and 3% tannin extract. The results showed that the additives modulated the silage microorganism composition. However, this was without affecting the silage’s fermentative quality and aerobic stability. All the silages recorded a pH below 4.2, which indicated well-fermented silage. The tannin extract suppressed the growth of undesirable bacteria, such as *Dysgonomonas*, *Gluconacetobacter* and *Clostridium* genera, while promoting desirable bacteria, such as *Lactobacillus* and *Weissella* genera, which were attributed to the silage quality. It is thus concluded that tannins can be strategically used as silage additives to modulate the microbial composition of silage and improve the silage quality by promoting the dominance of the desirable bacteria in the silage.

## 1. Introduction

Tannins are water-soluble polyphenolic compounds that are synthesised as mechanical defence mechanisms to prevent plants from being consumed by animals. Terrestrial plants synthesise two classes of tannins, which are condensed and hydrolysable tannins [1,2]. Condensed tannins are made up of flavan-3-ols and flavan-3,4-diols, while hydrolysable tannins are made up of gallotannins and ellagitannins [3]. Condensed tannins are the most ubiquitous polyphenols synthesised by terrestrial plants [1]. Tannins are characterised by several factors, including the ability to form complexes with proteins (bind proteins) and polysaccharides owing to the presence of hydroxyl phenolic groups [1,4,5]. In addition, tannins are antimicrobial and antiparasitic and have immunomodulatory properties [6]. Therefore, owing to their antimicrobial effects, tannin extracts have been used as silage additives to regulate proteolysis on ensiled high-protein forage materials such as alfalfa [7], *Moringa oleifera* and *Indigofera* leaves [8]. This shows that tannins have the potential to be used as silage additives to manipulate silage microorganisms and prevent nutrient loss due to fermentation. However, few studies have reported the use of tannins to improve silage quality by inhibiting undesirable bacteria while promoting desirable bacteria.

Over the years, ensiling has been a commonly practiced technology to preserve fresh forage materials for feeding animals during scarcity periods [9,10,11] because, compared with hay, silage loses very little nutritional value due to unfavourable weather conditions [12]. According to Bernardes et al. [13], silage-making is not limited by weather conditions to such an extent that silage can be made successfully in hot or cold regions. Moreover, the benefit of ensiling is bale technology, where storage can be accomplished without buildings [10]. Silage can be produced from different forage sources, such as grasses and legumes [14], sorghum [15], maize [16], tree fodder [7,11] and agro-industrial by-products [17,18]. Generally, ensiled forage material undergoes a fermentation process where lactic acid bacteria use water-soluble sugars to produce lactic acid [19]. Lactic acid production helps to rapidly reduce the pH value of ensiled materials to below 5 within the first three days after ensiling [12]. However, during the fermentation process, which is influenced by different microorganisms [20], the silage nutritive value may be reduced owing to the degradation of protein and the deamination of amino acids [7,21,22]. Subsequently, this leads to a low protein utilisation efficiency of the ensiled forage material by animals [23], indicating the need to modulate silage microorganisms to improve the silage quality.

Likewise, the whole maize plant has been commonly used worldwide to produce silage for animal feeding. According to Khan et al. [24], maize silage has become a significant component of dairy cow rations in recent decades because of attributes such as its high-biomass yield with suitable starches, good water-soluble carbohydrates, and low buffering capacity [25,26]. Moreover, maize crops have stable yields and good ensiling characteristics under various environmental and climatic conditions [17]. Generally, the whole maize plant is suitable for ensiling because it provides adequate energy from starches in the kernel fraction and effective fibre, mainly as a neutral detergent fibre from the stover fraction [27]. However, the complex process that involves the interaction between forage enzymes and several bacteria during the fermentation of the ensiled forage material influences the biochemistry of the silage [25,28]. Therefore, the fermentation of the ensiled material is a dynamic process, which includes a series of bacterial organisms that lead to changes in silage metabolites [7,29]. Bacteria are reported to play an essential role in the successful fermentation of ensiled materials [20,27] and, hence, determine the silage quality [30], indicating the need to study silage’s microbiology and the influence of additives on the bacterial composition of the silage during ensiling.

Other studies have reported that lactic acid bacteria (LAB) inoculants affect the silage microbiology by increasing the abundance of specific bacteria over others [20,31,32,33]. For example, the increase in abundance of *Limosilactobacillus* and *Lentilactobacillus* bacterial genera have been reported for silages treated with LAB inoculants during ensiling [20,33,34,35]. These changes in silage bacterial composition are associated with the improved quality [33,35,36] and aerobic stability of open silage [20]. The manipulation of the silage microorganisms’ compositional structure is critical for improving the silage quality and nutrient-use efficiency [35]. Therefore, the objectives of this study were to evaluate the effects of *Vachellia mearnsii* (formerly known as *Acacia mearnsii*) condensed tannin as a silage additive on the fermentative quality, aerobic stability, and modulation of microbial composition. It was hypothesised that the *Vachellia mearnsii*-condensed tannin could effectively modulate the microbial composition of whole-maize crop silage without having negative effects on the fermentative quality and aerobic stability.

## 2. Materials and Methods

### 2.1. Study Area and Maize Harvesting

The study was conducted at the Agricultural Research Council-Animal Production (ARC-AP), Irene farm in South Africa. The details of the study’s geographical location and climatic conditions are provided by Mpanza et al. [37]. The ethics review and approval were waived because the study did not include the use of animals. *Zea mays* that was used for mini silage making in this study was planted at the ARC-AP farm during November 2021. The whole-maize crop was harvested for silage making at the half-milk growth stage at a dry matter (DM) content of 36.9% in mid-February 2022. Feraboli 945 forage harvester (Fondata Nel, Cremona, Italy) was used to harvest and chop the whole-maize crop to a theoretical length of 2–3 cm. The chopped maize forage was divided into five equal portions of 4 kg each on a fresh mass basis. Harvesting of the whole maize plant, chopping, and ensiling were conducted within the same day.

### 2.2. Treatments and Silage Preparation

Five treatments were employed for the mini-silo study, which were as follows: maize forage alone without inoculant (i.e., LAB or tannin extract), referred to as negative control (T1), maize forage inoculated with LAB and 1% tannin extract (T2), maize forage inoculated with LAB, referred to as positive control (T3), maize forage inoculated with LAB and 2% tannin extract (T4), and maize forage inoculated with LAB and 3% tannin extract (T5). The tannin extract inclusion percentage was calculated per kilogram of maize forage on fresh matter bases. The tannin extract used in this study was donated by the UCL (Pty) Ltd. Company, based in Dalton in Kwa-Zulu Natal, South Africa. Tannin was extracted from *Vachellia mearnsii* (Black wattle) bark by the UCL Company, and it is usually referred to as condensed tannin or Mimosa extract, because the extract contains 66% condensed tannin on dry matter basis. Therefore, the 1%, 2%, and 3% tannin extract inclusion levels used on maize silage are equivalent to 6.6 g, 13.2 g, and 19.8 g of condensed tannin inclusion per kg of fresh maize forage, respectively.

The lactic acid bacteria (LAB) inoculant that was used in this study was Kem LAC HD Dry (Kemin Industries, South Asia (Pty) Ltd.), which hereafter will be referred to as LAB. The Kem LAC HD Dry is a white powder containing lactic acid-producing bacteria, including *Lactobacillus plantarum*, *Lactobacillus acidophilus*, and *Lactobacillus bulgaricus*. All these bacteria have the same concentration of 1.36 × 10^9^ Colony Forming Units per gram (CFU/g). The LAB inoculant helps in improving the silage fermentative quality by increasing the production of lactic acid, reducing proteolysis, controlling silage temperature, and enhancing aerobic stability and DM retention [38]. The Kem LAC HD Dry inoculant was purchased from OBARO (Pty) Ltd., Pretoria, in South Africa. The amount of LAB used as an inoculant in this study was based on a company recommendation of 1.5 g per ton of silage on a fresh mass basis.

Tannin extract powder was spread on the chopped maize forage and thoroughly mixed by hand throughout the maize forage. The LAB inoculant was dissolved in 20 mL of distilled water two hours before being used. The negative control treatment was sprayed with only 20 mL of distilled water to standardise the silage moisture content. After a thorough mixing of tannin extract with 4 kg maize forage per treatment, it was then sprayed with 20 mL distilled with or without LAB inoculant, hand mixed again and ensiled immediately using a 1.5 L glass jar. The forage was hand-pressed into each jar to reduce air as much as possible while creating an anaerobic condition suitable for good fermentation. Approximately 970 g of fresh maize forage was manually packed in a pre-weighed glass jar, with each treatment replicated three times. The jars that were used for ensiling were equipped with lids that can release the buildup gas during fermentation. Ensiled material was stored in a dark room at a temperature 23–25 °C, and fermentative quality, aerobic stability and microbial composition were determined after 75 days post ensiling.

### 2.3. Characterisation of Fresh Forage and Silage

On day zero (ensiling day), samples of about 80 g of fresh maize (three replicates per treatment) were taken. Each 80 g sample was divided into two equal portions; one portion (40 g) was mixed with 350 mL distilled water, vigorously shaken for 2 min, and kept in a fridge at 5 °C overnight. The mixture was filtered through Whatman No 1 filter paper the following day. The pH of the extract was determined with a pH meter (Thermo Orion Model 525, Thermo Fisher Scientific, Waltham, MA, USA). Another 40 g portion was oven-dried at 60 °C for 72 h to determine the dry matter content at ensiling. After 75 days of ensiling, glass jars with silage were weighed to estimate the weight loss per treatment owing to fermentation. Three glass jars of the same treatment were opened, the top 2 cm spoiled silage was discarded, and the rest was emptied into a 10 L bucket one at a time and thoroughly hand-mixed. To prevent cross-contamination, plastic hand gloves were used per treatment and discarded afterwards. Silage pH and dry matter were determined, as described above. In addition, about 15 g (two samples per treatment) of silage sample was taken into a plastic container, which was closed tightly and frozen at −80 °C until further used for DNA extraction (see Section 2.5 below for details). Dry matter recovery was determined by calculating the quotient between the DM at ensiling (i.e., day zero) and the DM of the silage (i.e., day 75). Dry matter content was determined by oven drying the samples of fresh maize (i.e., day zero) and silage (i.e., day 75) at 60 °C for 72 h, while weight losses owing to the fermentation of maize mini-silo were determined by calculating the difference between silage weights at day 75 and the weight of fresh forage at day 0. The weight at ensiling was determined by weighing the empty jars and jars filled with maize forage for ensiling. After 75 days of ensiling, before opening, the weight of jars with silage were recorded per treatment and the weight of jars alone after having been emptied of silage. The difference between the empty jar’s weight and jars filled with fresh forage or silage was used to estimate the weight of fresh maize forage at ensiling or the weight of the silage at opening, respectively.

### 2.4. Silage Aerobic Stability Test

To assess the aerobic stability of silage, an extra 100 g of silage sample at day 75 (three samples per treatment) was put loosely in a cleaned plastic container and exposed to oxygen for four days. A thermometer was inserted into the geometric centre of the silage to record the temperature in every 24 h for four days using a Brannan thermometer (CA25 5QE, Cleator Moor, Cumbria, UK). The silage was covered with nylon to prevent dust contamination while allowing airflow, as described by Li et al. [39]. Containers with silage were kept at a room temperature of 24 °C. Silage was considered not to be aerobically stable when the silage temperature rose 2 °C above room temperature within the four days of oxygen exposure.

### 2.5. Deoxyribonucleic Acid (DNA) Extraction and Sequencing Analysis

Microbial DNA was extracted using a Macherey-Nagel™ NucleoSpin™ DNA Stool kit (Macherey-Nagel, Düren, Germany) following the manufacturer’s guidelines, and DNA concentration was evaluated with Nanodrop 2000 (Thermo Electron Corporation, Waltham, MA, USA). The 16S Metagenomics Sequencing Library Preparation guide (Illumina, San Diego, CA, USA) was followed to perform 16S rRNA amplification and sequencing. The V3-V4 hypervariable regions were amplified using primers with adapters (Forward = 5′TCGTCGGCAGCGTCAGATGTGTATAAGAGACAGCCTACGGGNGGCWGCAG3′, Reverse = 5′GTCTCGTGGGCTCGGAGATGTGTATAAGAGACAGGACTACHVGGGTATCTAATCC3′). The PCR was conducted using the reaction procedures detailed by Mpanza et al. [37]. The purified PCR products were sequenced using the Illumina MiSeq platform and generated 300 bp paired-end reads. The raw reads were trimmed to remove low-quality sequences with Trimmomatic version 0.36. QIIME2 software version 2022.2 was employed for downstream analysis, pre-processing paired-end sequence reads using the DADA2 pipeline, including QC, denoising, merging sequences, and removing chimeric sequences. Sequences were clustered de novo, and sequences with ≥97% similarity were assigned to the same operational taxonomic units (OTUs). The OTU feature table was generated, and representative OTU sequences were aligned to the Greengenes database for taxonomic assignment.

### 2.6. Statistical Data Analysis

Data on pH, dry matter recovery, weight loss, and aerobic stability were subjected to one-way analysis of variance (ANOVA) using SAS version 9.0 (SAS Institute, Inc., Cary, NC, USA). The following statistical model was used:

Y*_ij_* = µ + ġ*_i_* + ε*_ij_*

where Y*_ij_* is a general observation, µ is a general mean, ġ*_i_* is the additives effects (*i* = LAB, tannin extract), and ε*_ij_* is the random error effect. Regarding a significant difference between treatment means, the PDIFF statement was used to compare the means. Significance was declared at *p* ≤ 0.05, and the tendency at 0.05 < *p* ≤ 0.1.

Bioinformatics data analysis on the microbial composition of silage was performed using RStudio with R core (version 4.2.2). Alpha diversity matrices (observed and Shannon) were calculated and tested for variances using the Kruskal–Wallis test. Beta diversity was calculated using distance matrices generated from unweighted UniFrac analysis. Principal coordinate analysis (PCoA) was conducted to explore the bacterial clustering of silage treatments as influenced by additives. A canonical correspondence analysis (CCA) was conducted to determine the relationships between the microbial compositions between different maize silage treatments. An analysis of similarities (ANOSIM) was conducted to evaluate similarity on bacterial composition between maize silage treatments influenced by additives. Lastly, a Van diagram was drawn to demonstrate common and unique bacterial operational taxonomic units (OUTs) between maize silage treatments as influenced by additives.

## 3. Results

### 3.1. Fermentative Characteristics of Maize Silage

The fermentative characteristics of maize silage were measured by determining silage pH, dry matter (DM), dry matter recovery (DMR), and weight loss (WL) on opening day (i.e., day 75). Table 1 showed the fermentation quality of maize silage treated with different additives. These results showed that the pH value at ensiling (day 0) was above 5, as was expected since no fermentation had occurred. It was noticed that treatment 5 had a significantly (*p* < 0.05) higher pH value compared to other treatments. However, it is worth noting that the pH of all silage treatments dropped to below 4.0 at day 75 after ensiling. Therefore, tannin extract as an additive did not have a negative effect on the fermentative characteristics of maize silage. However, it was observed that there was a significant (*p* < 0.05) variation in pH values among the treatments at day 75, with treatment 5 recording the highest pH value.

Furthermore, this study showed a significant (*p* < 0.05) variation in silage dry matter content. Treatments 4 and 5, treated with 2% and 3% of tannin extract, recorded the highest DM. It was observed that the tannin extract additive did not affect the dry matter recovery (DMR) of the silage; however, there was a tendency to improve DMR with a *p*-value of 0.0624. For treatments 2 and 4, silage recorded a 13.1% and 14.0% higher DMR, respectively, compared to silage in T1 (negative control), but it was not statistically significant (*p* > 0.05). A similar pattern was observed with silages in T3 and T5, which recorded a numerically higher DMR than the negative control, but it was not statistically significant (*p* > 0.05). The same pattern was also observed in weight loss (WL), owing to fermentation. Tannin extract as an additive did not affect the WL of maize silage, even though silages in T2 and T4 reduced weight loss by 68.7% and 67.2%, respectively, as compared to the negative control treatment (maize silage without additives).

### 3.2. Silage Aerobic Stability as Influenced by Additives

**Figure 1:**
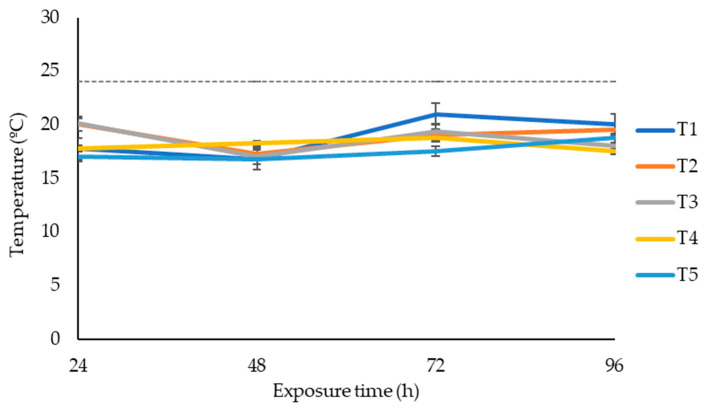
Temperature dynamics of maize silage treatments exposed to air over four days. T1, maize forage without LAB or tannin extract inoculant (negative control); T2, maize forage inoculated with LAB and 1% tannin extract; T3, maize forage inoculated with LAB only (positive control); T4, maize forage inoculated with LAB and 2% tannin extract; and T5, maize forage inoculated with LAB and 3% tannin extract. Dotted line represents the room temperature during aerobic exposure (24 °C).

After 75 days of ensiling, silage was subjected to aerobic stability. The effects of additives on maize silage aerobic stability are shown in Figure 1. The temperature changes in maize silage exposed to oxygen were recorded over four days. Silage treated with LAB (positive control) or LAB and tannin extract did not affect the silage aerobic stability within the four days of silage exposure to oxygen. None of the silage treatments recorded a temperature above the room temperature of 24 °C within the four days of silage exposure to oxygen.

### 3.3. Silage Microbial Composition as Influenced by Additives

**Figure 2:**
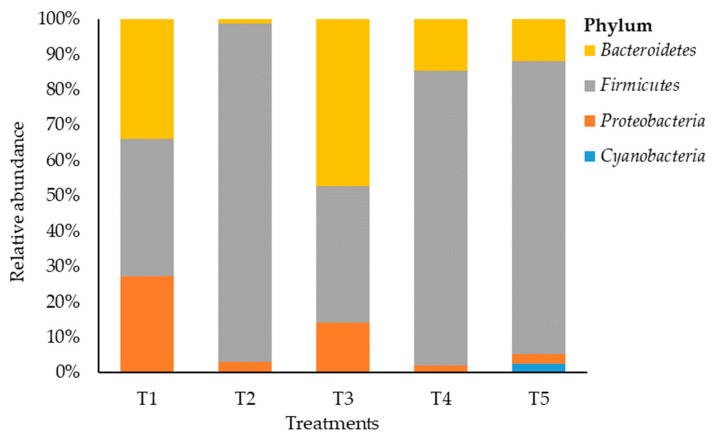
Relative abundance of bacteria on maize silage at the phylum level. T1, maize forage without LAB or tannin extract inoculant (negative control); T2, maize forage inoculated with LAB and 1% tannin extract; T3, maize forage inoculated with LAB only (positive control); T4, maize forage inoculated with LAB and 2% tannin extract; and T5, maize forage inoculated with LAB and 3% tannin extract.

A total of 46 OTUs were obtained from the samples. The bacterial composition was estimated across treatments, with 4 phyla detected at a relative abundance of ≥0.05% (Figure 2). Maize silage treated with no additive (negative control) showed that *Firmicutes* and *Bacteriodetes* phyla were dominant but none of the phyla reached 40% each, and the two phyla constituted 72.8% of the bacterial phylum abundancy that was detected. However, LAB-treated maize silage (positive control) showed a shift in bacterial dominancy to phylum *Bacteroidetes* with a relative abundance of 50.7%. On the other hand, maize silage treated with tannin extract additive (T2, T4, and T5) was dominated by phylum *Firmicutes*. The phylum *Firmicutes* recorded a relative abundancy of 97.2%, 83.7%, and 85.1% in T2, T4, and T5, respectively. This indicates an increase from 2.2 to 2.5 times the value of 38.85% recorded for *Firmicutes* in T1 (negative control) silage. *Proteobacteria* accounted for 27.2%, 3%, 15.2%, 2.1%, and 2.8% in T1, T2, T3, T4, and T5, respectively. Phylum *Cyanobacteria* was recorded only in T5 with a relative abundance of 2.6%.

**Figure 3:**
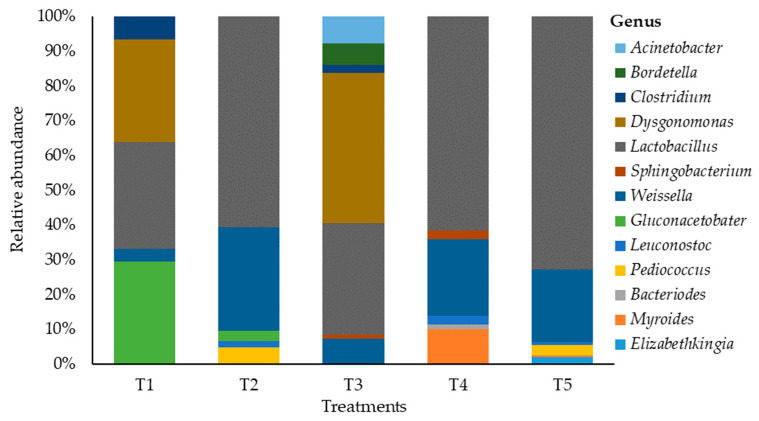
Relative abundance of bacteria on maize silages at genus level. T1, maize forage without LAB or tannin extract inoculant (negative control); T2, maize forage inoculated with LAB and 1% tannin extract; T3, maize forage inoculated with LAB only (positive control); T4, maize forage inoculated with LAB and 2% tannin extract; and T5, maize forage inoculated with LAB and 3% tannin extract.

Changes in the bacterial community on maize silage at the genus level as influenced by additive are shown in Figure 3. This study showed a shift in bacterial abundance at the genus level that was associated with the silage additive. The maize silage treated with no additives (negative control) was dominated by genera *Lactobacillus*, *Dysgonomonas*, and *Gluconacetobacter* with the relative abundances of 30.2%, 29.1% and 29.1%, respectively. Silage treated with LAB (positive control) showed a shift in bacterial dominancy from the genus *Lactobacillus* (35.2%) to genus *Dysgonomonas* (47.3%), and *Weissella* (7.9%) was the third dominating genus. In addition, treating maize silage with LAB during ensiling showed a negative effect on *Clostridium* bacteria while promoting *Dysgonomonas* bacteria. Hence, *Clostridium* abundancy was reduced by 0.4 times, whereas *Dysgonomonas* abundancy was increased by 1.6 times. This study showed that treating maize silage with tannin extract during ensiling promoted the dominancy of *Lactobacillus* bacteria, with abundancy levels of 63.7%, 63.5%, and 73.2% recorded in T2, T4, and T5 silages. This indicates an increase in more than double the value (30.2%) recorded in T1 (negative control) silage. The *Weissella* genus was the second dominant bacteria in silage treated with tannin extract, with the relative abundancies of 31.3%, 22.8%, and 21.1% recorded in T2, T4, and T5 silages. On the other hand, the tannin extract additive inhibited the establishment of genera *Clostridium* and *Dysgonomonas*, while promoting the dominancy of *Lactobacillus* bacteria in the silage. Other genera such as *Pediococcus* (5.1%, 2.6%, and 3%) and *Leuconostoc* (1.9%, 2.6%, and 0.9%) were recorded on silage treated with tannin extract (i.e., T2, T4, and T5).

**Figure 4:**
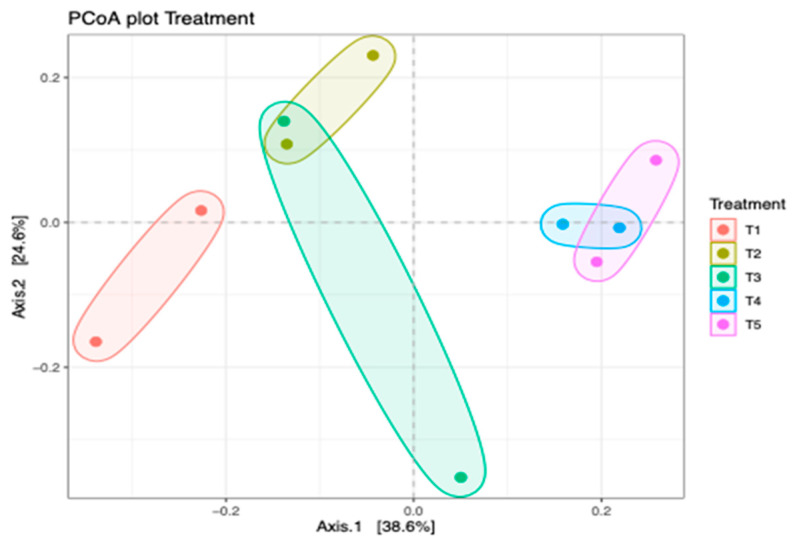
Principal coordinate analysis (PCoA) of the bacterial community in maize silage depicting treatment differences. T1, maize forage without LAB or tannin extract inoculant (negative control); T2, maize forage inoculated with LAB and 1% tannin extract; T3, maize forage inoculated with LAB only (positive control); T4, maize forage inoculated with LAB and 2% tannin extract; and T5, maize forage inoculated with LAB and 3% tannin extract.

There was no significant (*p* > 0.05) difference in the alpha diversity of the bacterial community in different silage treatments. Figure 4 shows the principal coordinate analysis (PCoA) of bacterial composition in maize silage as influenced by additives. The PCoA showed that axis 1 accounted for 38.6% variations of bacterial differences owing to negative control treatment (T1 silage), whereas bacterial differences between T2, T3, T4, and T5 silages accounted for 24.6% variations of axis 2. The PCoA showed that silage additives resulted in a variation in bacterial composition between the treatments. The bacterial composition of maize silage treated with no additives (negative control) was clearly separated from the bacteria from silages treated with either LAB or tannin extract additives. However, there was an overlap in bacterial clustering on silage treated with LAB (positive control) and silage treated with LAB and 1% tannin extract (T2). It was noticed that increasing the level of tannin extract inclusion as an additive in maize silage further promoted bacterial variation. This study showed that in treatments 4 and 5, bacteria were clustered separately from the rest of the treatments, even that of T2, although it was treated with tannin extract.

**Figure 5:**
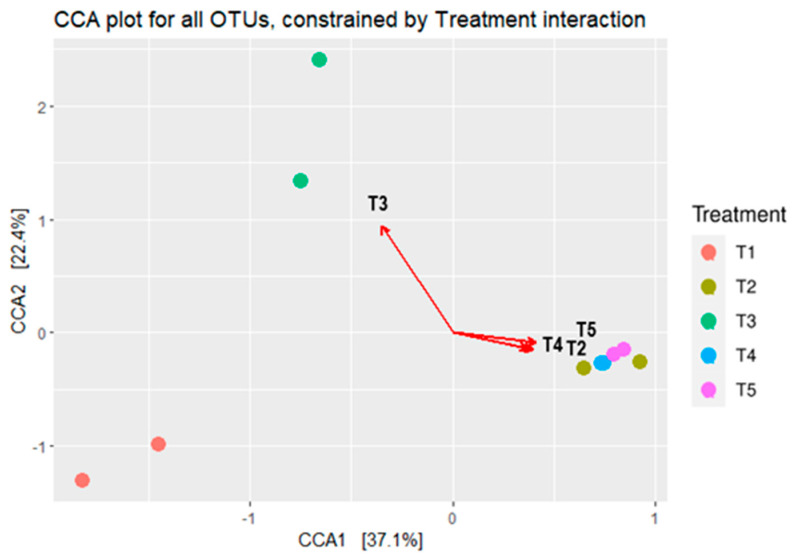
Canonical correspondence analysis (CCA) shows the microbial composition relationships between maize silage treatments. T1, maize forage without LAB or tannin extract inoculant (negative control); T2, maize forage inoculated with LAB and 1% tannin extract; T3, maize forage inoculated with LAB only (positive control); T4, maize forage inoculated with LAB and 2% tannin extract; and T5, maize forage inoculated with LAB and 3% tannin extract. Arrows show the relationships between the treatments.

The bacterial composition’s canonical correspondence analysis (CCA) in maize silages is depicted in Figure 5. The CCA showed a clear separation in silage bacterial composition between the treatments. The CCA showed that the bacterial composition of maize silage treated with no additive (negative control) and treated with either LAB or tannin extract as additives had no relationships with each other. On the other hand, silages that were treated with tannin extract showed a relationship to each other, and hence were grouped separately from the others.

**Figure 6:**
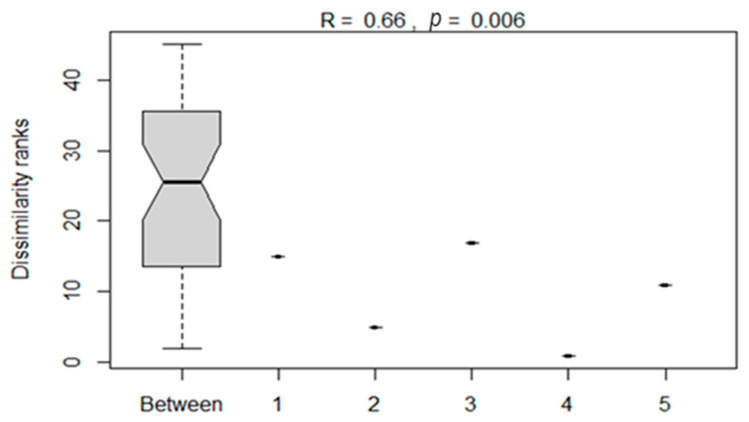
Analysis of dissimilarities in the bacterial composition of maize silages between treatments. Numbers 1–5 denote treatments (T1, maize forage without LAB or tannin extract inoculant; T2, maize forage inoculated with LAB plus 1% tannin extract; T3, maize forage inoculated with LAB only; T4, maize forage inoculated with LAB plus 2% tannin extract; and T5, maize forage inoculated with LAB plus 3% tannin extract).

The analysis of similarities between the bacterial compositions of silage treatments as influenced by additives is presented in Figure 6. The ANOSIM showed a significant (*p* < 0.05) dissimilarity between bacterial compositions of maize silages subjected to different treatments. This showed that silage additives influenced the variation in bacterial composition on maize silage.

**Figure 7:**
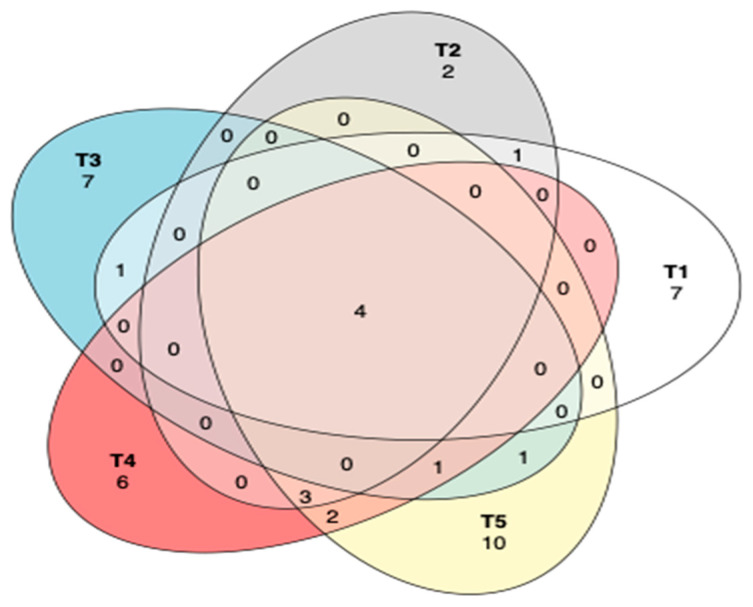
Venn diagram to describe the common and unique OTUs among silage treatments. T1, maize forage without LAB or tannin extract inoculant (negative control); T2, maize forage inoculated with LAB and 1% tannin extract; T3, maize forage inoculated with LAB only (positive control); T4, maize forage inoculated with LAB and 2% tannin extract; and T5, maize forage inoculated with LAB and 3% tannin extract.

The common and unique operational taxonomic units (OTUs) between different treatments of maize silage as influenced by additives are shown in Figure 7. A Venn diagram showed that there were only four OTUs that were shared among all the silage treatments, while most OTUs were unique to each treatment. However, silage in treatment 2 showed only two unique OTUs. Tannin-treated silages shared three OTUs, while treatments 4 and 5 shared two OTUs.

## 4. Discussion

### 4.1. Fermentative Characteristics and Aerobic Stability of Maize Silage

The pH value of the silage is used as one of the parameters to judge the fermentative quality and as an indicator of well-preserved silage. A well-fermented silage is recommended to have a pH value of 4.2 or below [25]. This is because, during fermentation, organic acid (mainly lactic acid) is produced by lactic acid bacteria, which helps in a drastic reduction of the pH to below 5 within the first three days of ensiling [12,19,40]. In this study, the pH values at day zero (ensiling day) were above 5, while at day 75 post-ensiling the pH values dropped below 4.2 (Table 1). Therefore, in this study, all treatments produced sufficient lactic acid required to reduce the pH, indicating a well-preserved silage with a good fermentative quality. The pH values recorded on different silage treatments in this study were in the range of adequately fermented silage, which is below 4.2 [34]. In addition, a pH value below 4.2 helps to reduce chances for the establishment of the undesirable microorganisms in the silage [41,42]. Therefore, it is safe to say that *Vachellia mearnsii* tannin extract did not interfere with the silage fermentation characteristics of maize silage; hence, maize silage treated with tannin extract fermented well. This is because silage pH is considered the critical indicator of the fermentation quality of the ensiled forage material [11] and for adequately preserved silage [43]. The addition of *Vachellia mearnsii* tannin extracts had no influence on the stability of the fermentation process.

The dry matter (DM) content of maize at ensiling ranged from 34.7% to 40.2%, which was within the recommended range (28% to 40%) for forage at ensiling [44]. On the opening day (day 75), the silage had a DM content ranging from 31.1% to 36.5%, above 28%, the minimum recommended DM for silage [44,45]. Therefore, this indicates that maize silages were preserved well [44]. However, maize silage from treatments 4 and 5 had a significantly higher DM percentage as compared to other treatments (Table 1). This study showed that treating maize during ensiling with tannin extract had no significant effect on the dry matter recovery and weight loss of silage owing to fermentation, even though the tannin-treated silage showed numerically higher DMR and lower WL. Silage dry mater recovery coupled with low weight loss indicates a low moisture content, and such silage is reported to maintain a low temperature during aerobic exposure [46]. This agrees with our results for aerobic stability; all treatments recorded temperatures below the ambient temperature over the four days of oxygen exposure (Figure 1). However, these results must be interpreted with caution, owing to the fact that silage was exposed to oxygen only for four days and we do not know how long the silage would have maintained aerobic stability if it were exposed for longer than four days. The pattern of silage temperature exposed to oxygen could have been explained better if microbial composition and the pH value of aerobically exposed silage were recorded. The silage temperature pattern would have been associated with microbial composition and the pH values of the silage after exposure to oxygen. On the other hand, reducing the weight loss of the ensiled forage materials is regarded as a biological benefit, as it improves silage production [14]. However, in this study, tannin inclusion levels of 1% and 2% showed a tendency to improve silage production by reducing the weight loss of maize silage.

Silage fermentative characteristics (i.e., pH, short-chain fatty acids, DMR and weight loss) are good indicators of well-preserved silage; however, this does not necessarily determine the quality of silage. Therefore, it is essential to characterise what is in the silage to determine the quality. On the other hand, microbes have been reported as drivers of silage quality depending on the substrates present during ensiling [27,30]. Therefore, the molecular characterisation of silage microorganisms is essential to understand the microbial composition of silage, as the dominant bacteria in silage has been reported to determine the quality of silage [30,47]. Therefore, genomics technology offers an opportunity to study the microbiological aspects of the ensiled forage material to determine the silage microbial composition. Subsequently, in this study, 16S ribosomal RNA gene sequencing was conducted in maize silage after 75 days of ensiling to evaluate the effects of additives on microbial composition.

### 4.2. Silage Microbial Composition as Influenced by Additives

The microbial characterisation of maize silage revealed that additives influenced the bacterial composition of the silage (see Figure 5, Figure 6 and Figure 7). The high relative abundance of a few dominant phyla (*Bacteroidetes*, *Firmicutes*, and *Proteobacteria*) recorded in this study was attributed to a decrease in bacterial composition, which allied with the additives used during ensiling. Results reported in this study showed that silage additives led to a shift in bacterial dominance between the treatments. Applying LAB inoculant (T3) on maize silage during ensiling favoured the dominancy of phylum *Bacteroidetes* followed by phyla *Firmicutes* and *Proteobacteria*. On the contrary, Jaipolsaen et al. [48] and Dong et al. [49] reported *Firmicutes* bacteria as the dominant phylum on maize silage treated with LAB inoculant during ensiling. However, other studies reported *Proteobacteria* as the dominant phylum on silage produced from different forages while treated with LAB inoculant during ensiling [20,27,41]. The variation in bacterial dominance reported by various studies can be attributed to the LAB substrates used as an additive and the forage material used for silage production. The current study further showed that including tannin extract as a silage additive modulated the microbial composition of the silage, favouring the dominance of phylum *Firmicutes*. These results agree with other studies that reported the dominancy of phylum *Firmicutes* on silage produced from different forage materials [41,50,51,52,53,54]. Phylum *Firmicutes* constituted from 84% to 97% of the bacterium detected on maize silage in T2, T4, and T5, while in T1 and T3 maize silage, phylum *Firmicutes* constituted 39% and 42% of the detected bacteria, respectively. The dominance of *Firmicutes* in silage was reported as an indicator of well-fermented and good-quality silage [55], and this is because *Firmicutes* bacteria are reported to have the potential to secrete various enzymes under anaerobic conditions [55], which are involved in degrading the lignin content of the ensiled forage [56,57].

A low pH in silage indicates well-preserved silage and prevents undesirable bacterial growth [54]; this was observed in the current study, as all treatments recorded a pH below 4.0. However, the microbial characterisation of these silages using genomic technology analysis revealed the presence of undesirable bacteria in T1 (negative control) and T3 (positive control) silages. Thus, this study detected the bacteria *Dysgonomonas*, *Gluconacetobacter*, and *Clostridium*, which are classified as the undesirable genera in silage. In the T1 silage, these bacteria accounted for about 65% of the total detected genera. In T3 silage, genera *Dysgonomonas* and *Clostridium* were detected, and constituted about 50% of the detected bacteria. The presence of these undesirable genera in silage compromises the silage quality despite its fermentative quality [41,58]. The *Dysgonomonas* genus was reported to occur in response to circumstances rather than naturally [58]; therefore, it is necessary to study its presence in silage. The genus *Gluconacetobacter* is reported to favour acetic acid production, which increases silage pH [41]. *Clostridium* bacteria are reported to be involved in the production of alcohol in silage [59]. Furthermore, *Clostridium* bacteria decompose protein into ammonia nitrogen and lead to protein loss from the silage [60]. Therefore, the presence of *Clostridium* bacteria in silage is associated with several problems when such silage is fed to animals; these include nitrogen pollution due to high ammonia [61], ketonemia, and a reduction of milk production in cattle [62].

On the other hand, tannin-treated silages were dominated by the genus *Lactobacillus* bacteria, which is associated with well-preserved silage [42]. *Lactobacillus* is recorded as an essential microorganism during ensiling due to its ability to control lactic fermentation [41,63]. Therefore, in high-quality silage, *Lactobacillus* is the predominant bacteria [64]. Likewise, in this study, *Lactobacillus* was the most abundant bacteria in tannin-treated silage. *Weissella* bacteria was the second dominant genus in maize silage treated with tannin extract, accounting for 31.3%, 22.8%, and 21.1% in T2, T4, and T5, respectively. *Lactobacillus* and *Weissella* bacteria are reported to influence lactic fermentation by producing lactic acid during ensiling [33]. Hence, these two genera are reported to contribute significantly in reducing silage pH, particularly in the early phase of silage, and also in sustaining low silage pH [36,50]. In addition, *Weissella* bacteria are reported to convert soluble carbohydrates into carbon dioxide and water during silage fermentation [42]. The dominance of *Lactobacillus* and *Weissella* genera reported in this study on maize silage treated with tannin extract concurs with the result reported by [60].

The analysis of PCoA and CCA showed that additives, either LAB or tannin extract, played a critical role in the bacterial community structure of the silage. The CCA clearly showed that treating maize silage with condensed tannin extract during ensiling favours desirable bacteria (i.e., *Lactobacillus* and *Weissella* from phylum *Firmicutes*) which are critical for silage quality. ANOSIM also showed significant (*p* < 0.006) dissimilarities between the bacterial structure of maize silage as influenced by additives. This confirms that silage additives used in this study modulated the microbial composition of the silage. A Venn diagram also showed uniqueness in the OTUs, as influenced by silage treatments (additives). A well-preserved silage was reported to be dominated by phylum *Firmicutes* and genera *Lactobacillus* and *Weissella* bacteria [36]. A similar pattern has been observed in the present study, where maize silage treated with tannin extract improved silage quality by inhibiting the growth of the undesirable bacteria.

## 5. Conclusions

The pH values below 4.2 recorded on silages in this study showed that silage fermented well, despite the tannin additive. Treating silage with an additive during ensiling modulated the silage’s bacterial composition without negative effects on silage fermentative quality. However, *Vachellia mearnsii* tannin extract additive led to a shift in bacterial dominancy from phylum *Bacteroidetes* to phylum *Firmicutes*, which is a good indication of well-preserved silage. Treating maize silage at ensiling with *Vachellia mearnsii* condensed tannin inhibited the growth of undesirable bacteria (genera *Dysgonomonas*, *Gluconacetobacter*, and *Clostridium*) while promoting the growth of desired bacteria (genera *Lactobacillus* and *Weissella*). High-quality silages are dominated by genus *Lactobacillus* bacteria, and this study recorded this bacteria as the most abundant on silages treated with tannin extract. Therefore, this study showed that condensed tannin can be strategically used as a silage additive to improve silage quality without compromising the fermentative characteristics of the silage. Further study is required to determine the effects of feeding such silage on animal feed intake, digestibility, rumen modulation, methane emission, blood metabolites, and growth performance.

## Figures and Tables

**Table 1:** Effects of tannin additives on fermentative quality of maize silage.

Treatments	pH at Day 0	pH at Day 75	DM% at Day 75	DMR%	WL%
T1	5.13 ^b^	3.52 ^c^	31.1 ^b^	82.4	7.0
T2	5.15 ^b^	3.53 ^c^	33.3 ^b^	93.2	2.2
T3	5.16 ^b^	3.55 ^b^	32.7 ^b^	89.3	3.9
T4	5.17 ^b^	3.52 ^c^	36.4 ^a^	93.8	2.3
T5	5.29 ^a^	3.60 ^a^	36.5 ^a^	87.2	4.8
SEM	0.017	0.005	0.825	2.63	1.13
*p*-values	0.0004	<0.0001	0.0032	0.0624	0.0674

DM, dry matter; DMR, dry matter recovery; WL, weight loss; T1, maize forage without LAB or tannin extract inoculant (negative control); T2, maize forage inoculated with LAB and 1% tannin; T3, maize forage inoculated with LAB only (positive control); T4, maize forage inoculated with LAB and 2% tannin; and T5, maize forage inoculated with LAB and 3% tannin. SEM, standard error of the mean. ^a–c^ Superscript letters in a column mean significant difference at *p* ≤ 0.05.

## Data Availability

The sequences presented in this study were deposited at the National Center of Biotechnology Information (NCBI) Sequence Read Archive (SRA) database under Bioproject PRJNA976900 and can be accessed via this link: https://www.ncbi.nlm.nih.gov/sra/PRJNA976900 (accessed on 4 October 2023).
